# Molecular Evolution of a Novel Family of Putative Calcium Transporters

**DOI:** 10.1371/journal.pone.0100851

**Published:** 2014-06-23

**Authors:** Didier Demaegd, Anne-Sophie Colinet, Antoine Deschamps, Pierre Morsomme

**Affiliations:** Institut des Sciences de la Vie, Université Catholique de Louvain, Louvain-la-Neuve, Belgium; Simon Fraser University, Canada

## Abstract

The UPF0016 family is a group of uncharacterized membrane proteins, well conserved through evolution and defined by the presence of one or two copies of an E-Φ-G-D-(KR)-(ST) consensus motif. Our previous results have shown that two members of this family, the human TMEM165 and the budding yeast Gdt1p, are functionally related and might form a new group of cation/Ca^2+^ exchangers. Most members of the family are made of two homologous clusters of three transmembrane spans, separated by a central loop and assembled with an opposite orientation in the membrane. However, some bacterial members of the family have only one cluster of transmembrane domains. Among these ‘single-domain membrane proteins’ some cyanobacterial members were found as pairs of adjacent genes within the genome, but each gene was slightly different. We performed a bioinformatic analysis to propose the molecular evolution of the UPF0016 family and the emergence of the antiparallel topology. Our hypotheses were confirmed experimentally using functional complementation in yeast. This suggests an important and conserved function for UPF0016 proteins in a fundamental cellular process. We also show that members of the UPF0016 family share striking similarities, but no primary sequence homology, with members of the cation/Ca^2+^ exchangers (CaCA) superfamily. Such similarities could be an example of convergent evolution, supporting the previous hypothesis that members of the UPF0016 family are cation/Ca^2+^ exchangers.

## Introduction

In our recent work, we described two homologous membrane proteins, TMEM165 in human and Gdt1p in the budding yeast, as putative Ca^2+^ transporters. TMEM165 deficiency has been shown to cause a new type of Congenital Disorder of Glycosylation (CDG) [1], a family of inborn metabolic diseases affecting the glycosylation pathway. We demonstrated that TMEM165 is localized to the Golgi and lysosomes of HeLa cells, which is consistent with the presence of typical lysosomal targeting sequences [2]. Furthermore, mutations associated with CDG patients caused mis-localization of TMEM165 [2] and enhanced acidification of lysosomes [3].

In parallel, experiments in yeast demonstrated that Gdt1p localized to the *cis*- and *medial*-Golgi, with a pattern similar to the yeast Ca^2+^/Mn^2+^ ATPase, Pmr1p [3]. Pmr1p and Gdt1p are both involved in sensitivity to high-Ca^2+^ concentrations, but we found that growth of the *gdt1Δ/pmr1Δ* double deletant was more severely reduced in high-Ca^2+^ concentrations than with either single deletant. Either protein can partially compensate for loss of the other and allow reduction of the cytosolic Ca^2+^ concentration, which is essential for cell survival in the presence of high external Ca^2+^ concentrations. Indeed, Ca^2+^ is an essential intracellular messenger and its cytosolic concentration has to be maintained at very low levels (typically 50–200 nM) [4]. These results indicated that Gdt1p and Pmr1p can each provide protection from high-Ca^2+^ stress, but via two distinct pathways. The Gdt1p-dependent pathway is thus a hitherto undescribed Ca^2+^ uptake system localized in the yeast Golgi apparatus [3]. The expression level of TMEM165 has been recently shown to increase 25 fold during lactation process in mammals supporting a role of this transporter as a contributor to mammary Golgi Ca^2+^ transport [5]. These results were therefore consistent with the suggestion that Gdt1p and TMEM165 might be Ca^2+^ transporters, and that the glycosylation defects observed in TMEM165-deficient patients might be a consequence of disturbed Ca^2+^ regulation in the Golgi apparatus.

Gdt1p and TMEM165 belong to a well conserved family of membrane proteins called UPF0016 (*Pfam accession number*: PF01169) and for which very little information is available. According to the database, members of this family are found in nearly all organisms (prokaryotes and eukaryotes) and possess one or two copies of an E-Φ-G-D-(KR)-(TS) consensus pattern (where Φ can be any hydrophobic residue). In this paper, we describe a detailed bioinformatic analysis of this family of proteins. We show that prokaryotic members of the family are particularly rich in evolutionary states. Indeed, they are found either as single-domain proteins, containing one consensus motif and three predicted transmembrane domains, or as two-domain (fusion) proteins, containing two homologous domains with opposite (antiparallel) membrane orientation. These two-domain proteins are likely to result from a duplication event. Moreover, genes coding for single-domain proteins are found in the genomes either as singletons or as pairs, directly adjacent on the chromosome. Analysis of these members allows us to retrace the evolutionary history of the family and adds more evidence to the current ideas explaining the appearance of two-domain membrane proteins [6], [7].

We also describe the specializations acquired by the eukaryotic members of the family. Notably, our analysis highlights the fact that the predicted topology and key features, but not the primary sequence of the family members are strikingly similar to those of the cation/Ca^2+^ (CaCA) superfamily [8]. Consistent with our previous results, this study supports the hypothesis that the members of the UPF0016 family may function as cation/Ca^2+^ exchangers, analogous to the CaCA supefamily of exchangers.

## Results and Discussion

### The UPF0016 Family can be Divided into Twelve Subfamilies

Members of the *Unknown Protein Family*, UPF0016 (*Pfam accession number*: PF01169), are extremely well conserved across kingdoms and species. Indeed, they are found in all eukaryotes and many bacteria (except *e.g.* in Lactobacillales and Bacillales) and archae. They are defined by the presence of one or two copies of an E-Φ-G-D-(KR)-(TS) consensus pattern (where Φ can be any hydrophobic residue).

**Figure 1:**
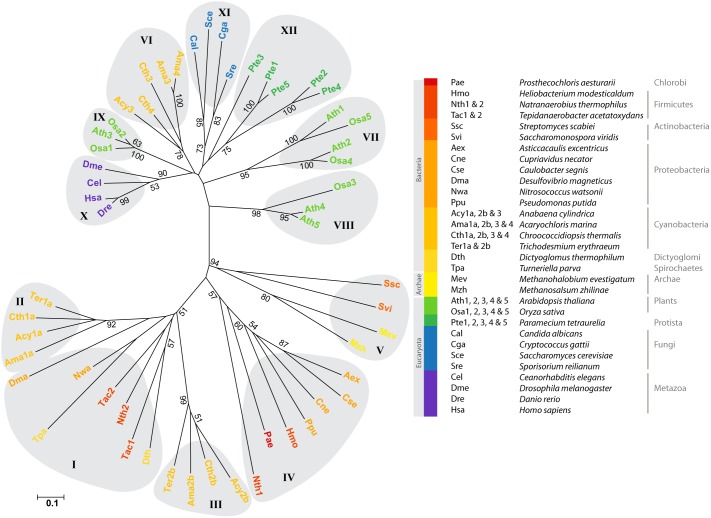
Phylogenetic tree of the UPF0016 family. The tree was constructed using the neighbor-joining method. It is drawn to scale with branch lengths measured as number of substitutions per site. Different taxonomic groups are represented by different colors, while different UPF0016 subfamilies are delimited by grey areas and numbered from I to XII. Bootstrap values (after 1000 iterations) higher than 50 are indicated.

To provide insight into the evolution of this family and draw a phylogenetic tree, sequences were arbitrarily selected from fully sequenced genomes. The main criterion for inclusion was to ensure the widest diversity of organisms. When applicable, all of the paralogs present in a given species were incorporated to the set of sequences. This survey was performed in December 2013 using PSI-BLAST with default parameters on the NCBI central database. Based on a final set of 149 sequences (Table S1), two different phylogenetic trees were constructed with prokaryotic and eukaryotic sequences, respectively (Fig. S1 & S2). For greater clarity, a simplified but global tree was also constructed from a restraint set of 55 sequences (Fig. 1).

Different subfamilies directly appear on the phylogenetic tree. Several attempts were made with different alignment methods (ClustalW, Muscle) or phylogenetic approaches (Neighbor-Joining, maximum likelihood) and, in all cases, the groups were identical, although the layout of the subfamilies relative to each other varied slightly. The major separation logically appears between prokaryotic (subfamilies I to VI) and eukaryotic (subfamilies VII to XII) sequences. However, the cyanobacterial sequences forming the subfamily VI appear to be more closely related to one of the plant groups, subfamily IX. Moreover, plants, which typically possess 2 to 5 paralogs per genome, also display the highest diversity.

A global alignment realized with one member of each subfamily (Fig. S3) highlights the similarities and differences found amongst the members of the UPF0016 subfamily. The two most conserved regions correspond to the two conserved motifs. The six putative transmembrane spans, predicted from the global alignment using TMAP [9], also correspond to more conserved regions, indicating that they could play an important role in the folding, stability, localization and/or activity of the proteins.

When present, the central hydrophilic loop is more heterogenous, but always contains several acidic residues. The presence of acidic motifs is common in divalent cation-binding proteins. For instance, members of the Ca^2+^-binding protein superfamily bind this cation using a characteristic ‘EF hand’ motif in which the majority of the coordination groups are provided by the side-chain carboxyl group of negatively charged acidic residues [10]. Similarly, the cardiac Na^+^/Ca^2+^ exchanger is regulated by the binding of Ca^2+^ to its intracellular domain. This domain contains two binding sites in which the main contributors to the Ca^2+^ binding are acidic segments [11].

The highest variability is found at the level of the N-terminus. Its length varies from one subfamily to another, and no obvious conservation appears between subfamilies. Notably, most prokaryotic proteins as well as the plant subfamily VIII lack any N-terminal extensions. These features will be discussed in more detail in the following sections.

### Evolution of an Antiparallel Two-domain Membrane Protein

Eukaryotic members of the UPF0016 family contain two homologous domains that are predicted to adopt opposite orientations in the membrane. The two domains probably arose from an ancient gene duplication event. Compared to eukaryotes, prokaryotic members of the family are found in more diverse forms: **singleton** genes coding for proteins of about 100 residues, **pairs** of genes adjacent on the chromosome and each encoding a slightly larger protein, and genes containing an internal duplication (**fusions**) and encoding two-domain proteins of approximately 200 residues. Members of the UPF0016 family are rather common in bacteria. They can be found in every phylum except Bacillales and Lactobacillales. In most of the phyla, they are found either as singletons or as fusions. Cyanobacteria are the only organisms in which paired genes are present, and the genes are always next to each other on the chromosome. Furthermore, paralogs are often observed (in about 20% of the selected species, when pairs are not considered as paralogs) as a probable result of multiple gene duplication events and/or horizontal gene transfer.

The two-domain members of the UPF0016 family are predicted to adopt a topology in which the domains are antiparallel in the membrane. Crystal structures have revealed an unexpected number of membrane proteins whose N- and C- terminal domains are related by a quasi-two-fold symmetry axis, either perpendicular to (parallel topology) or in the plane of the membrane (antiparallel topology) [6], [12]. Examples of proteins having antiparallel domains include the aquaporin family [13], the Na^+^-leucine transporter LeuT [14], the bacterial preprotein translocase subunit SecY [15], and the bacterial Na^+^/H^+^ exchanger [16]. All of these proteins are involved in transport of different materials across membranes. To explain the appearance of this kind of membrane protein, it is assumed that they evolved from an ancestral singleton gene encoding a ‘dual-topology’ protein, *i.e.* a protein able to insert in membranes in both orientations with similar likelyhood [6], [7]. This ancestral protein was then likely to associate into an antiparallel homodimer (or higher oligomer). Examples of such proteins have already been described, especially by an analysis of proteins in the small multidrug-resistance (SMR) family [17]. Among this family, the best characterized protein is EmrE, a multidrug transporter from *Escherichia coli*, which has been shown to function as a homodimer of a small four-transmembrane protein [18].

**Figure 2:**
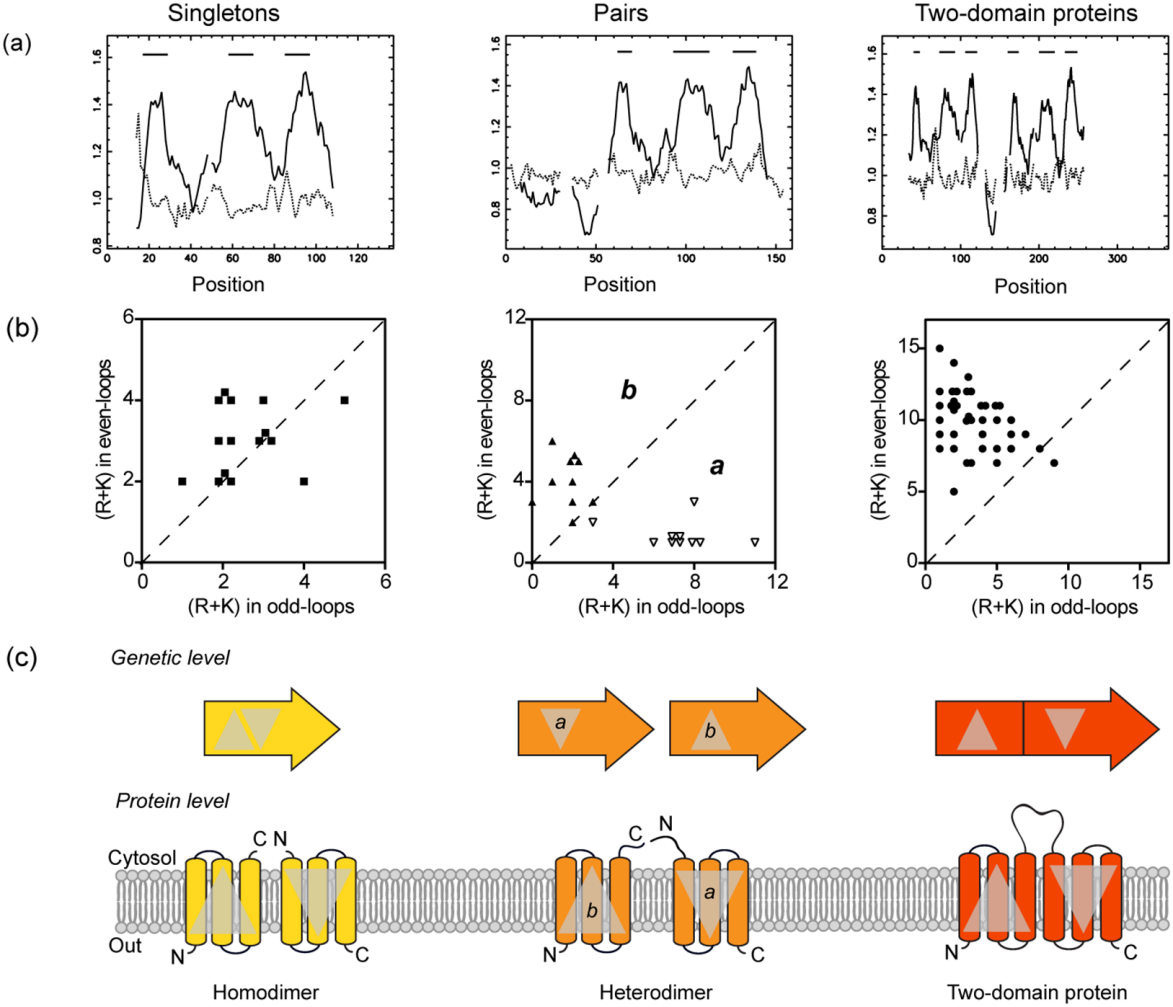
Illustration of the evolutionary intermediates found among prokaryotic UPF0016 members. (**a**) Topology of each subgroup predicted using TMAP based on multiple alignments obtained with the Muscle algorithm. A plot of the propensities to form the middle (plain line) or the end (dotted line) of the transmembrane regions is given; bars are displayed in the plots above the regions predicted to form transmembrane spans. (**b**) Distribution of positive charges (R+K) in the odd-loops (same side of the membrane as the N-terminus) plotted against the distribution of positive charges in the even-loops. Singletons (▪), pairs a (▿), pairs b (▴), and fusions (•). (**c**) Model of organization, at the genetic and protein levels, of the prokaryotic UPF0016 members. Inspired from [12].

To assess the topology of prokaryotic UPF0016 members, we first predicted the number of transmembrane domains in each subgroup using the TMAP software on a multiple alignment obtained via Muscle. As expected, the singletons and pairs were each predicted to have three transmembrane domains, while the fusions were predicted to have six (Fig. 2a). In bacteria, the insertion of proteins in the membrane follows the ‘positive inside’ rule, where the cytosolic loops of membrane proteins are generally more positive than the periplasmic loops, as a consequence of a bias in the distribution of positively (Arg, R, and Lys, K) but not negatively (Asp, D, and Glu, E) charged residues [19]. In other words, if the total number of positive charges (R+K) is smaller in the loops predicted to be on one side of the membrane compared to the loops predicted to be on the other side of the membrane, then the latter are likely to face the cytosol. When these numbers are equivalent, the protein is supposed to have no preferential orientation in the membrane, and could therefore adopt a dual-topology.

To apply this rule to prokaryotic UPF0016 family members, we counted the number of positive charges (R+K) located in the hydrophilic loops defined by the consensus topology model extended by five residues on each side. Positives charges in the odd-loops (same side of the membrane as the N-terminus) or the even-loops were counted and plotted (Fig. 2b). For the singletons, the points mainly follow the diagonal, meaning that there is only a weak (R+K) bias between the odd- and even-loops. Hence, these singleton proteins are equally likely to insert into the membrane in either orientation. They could, therefore, associate into homodimers to form an active complex (Fig. 2c).

**Figure 3:**
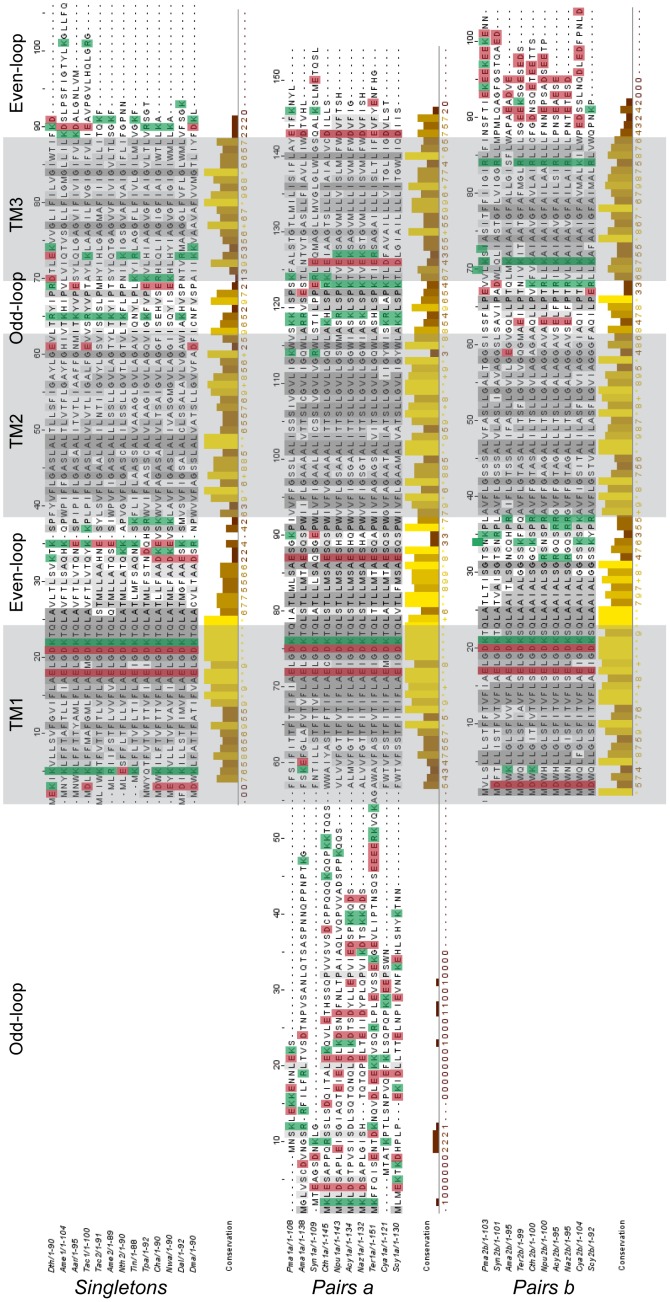
Repartition of the positive and negative charges within the products of prokaryotic singletons and paired genes. The sequences were aligned with the Muscle algorithm and the transmembrane domains predicted using TMAP (shaded in grey). The resulting alignments were visualized with Jalview: the conservation histogram is a quantitative annotation which measures the number of conserved physico-chemical properties conserved for each column. All of the positively charged residues (K+R) are highlighted in green, and the negatively charged residues (D+E) in red.

Within the pairs, each partner tends to belong to one of two groups. In the first, the data points lie above the diagonal (positively charged residues concentrated in the odd-loops), in the second they lie below (positively charged residues concentrated in the even-loops). Surprisingly, this separation occurs according to the position of the genes on the chromosome: all genes located on the 5′ position within a pair (*pairs a*) are found below the diagonal, while the genes located on the 3′ position (*pairs b*) are above the diagonal. Moreover, most of the first genes from each pair present a longer N-terminal extension which, according to the positive inside rule, should locate in the cytosol. In contrast, the C-terminus of the second members of each pair is longer and predicted to be cytosolic (Fig. 2c & Fig. 3). After the formation of the heterodimer, we suggest that those long cytosolic extensions may associate. This putative assembly of the N and C-terminal tails is also rich in acidic (negatively charged) residues. According to the positive inside rule, these acidic residues do not contribute to the orientation of the protein in the membrane.

Finally, all of the data points for the fusion proteins are above the diagonal, indicating that the even-loops present more positive charges. The central loop should thus face the cytosol in every ortholog present in our dataset (Fig. 2b & c). As noticed for the pairs, this central loop, which corresponds to the interface between both homologous domains, is also rich in acidic residues.

Similarly, members of a bacterial family of five-transmembrane proteins, the DUF606 family, have recently been shown to be especially rich in evolutionary states [12], [17]. These genes were also found as singletons, pairs, or two-domain fusions, and the authors suggested similar conclusions concerning their orientations in the membranes. To our knowledge, the DUF606 and UPF0016 families are the only examples displaying such a structural diversity.

### Experimental Confirmation of an Evolutionary Route to an Antiparallel Two-domain Protein

**Figure 4:**
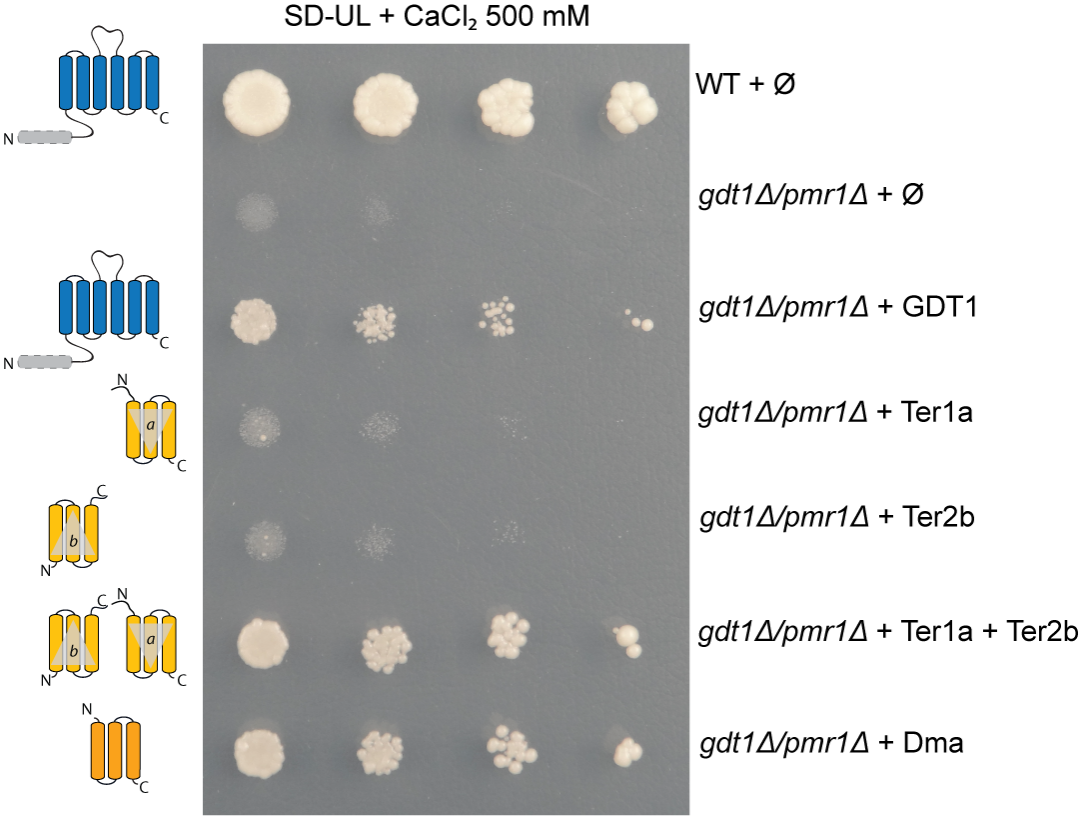
The singletons and paired genes (expressed simultaneously) are able to complement the absence of their yeast ortholog (Gdt1p). The different strains were precultured in minimal SD-UL medium to an OD_600_ of 0.3, and then serial tenfold dilutions were dropped onto solid SD-UL medium supplemented with 500 mM CaCl_2_ and incubated at 28°C for 10 days. All of the genetic constructions were in a pRS316 or pRS315 plasmid under the control of the pTPI promoter, and transformed into the *pmr1Δ/gdt1Δ* mutant. Φ represents the empty plasmid(s). This complementation assay demonstrates that a singleton gene (Dma) expressed alone or paired genes (Ter1a and Ter2b) expressed together are able to restore the growth of the *pmr1Δ/gdt1Δ* mutant in the presence of high Ca^2+^ concentrations. The paired genes expressed separately do not complement the absence of Gdt1p, confirming interdependency.

The suggestion that UPF0016 proteins evolved into two-domain single-gene proteins from gene duplication of proteins with ambiguous topologies, via pairs of genes encoding proteins with opposing topologies, lends itself to experimental complementation analysis in yeast. Yeast lacking their UPF0016 ortholog, Gdt1p (*Sce* in Fig. 1), show an increased sensitivity to high Ca^2+^ concentrations [3], [20]. This sensitivity is exacerbated when the Golgi-localized Ca^2+^-ATPase Pmr1p is absent [3]. If our analysis were correct, expression of a singleton gene should restore normal growth of the *gdt1Δ* or *pmr1Δ/gdt1Δ* mutant. In contrast, expression of each member of a pair separately might not allow the formation of a functional complex and should not therefore restore growth. Co-expression of both genes should allow complementation. For this experiment, we chose one singleton protein which was well located on the diagonal in Fig. 2a (*Dma*, from *Desulfovibrio magneticus*) and two members of a pair which were clearly distinct from the diagonal (Ter1a & Ter2b, from *Trichodesmium erythraeum*). After codon optimization, the corresponding genes were transformed into the *pmr1Δ/gdt1Δ* yeast mutant and the transformants dropped on high Ca^2+^-containing medium to analyze their growth. Figure 4 shows that the expression of *Dma* or *Ter1a* and *Ter2b* together is sufficient to restore growth. However growth in presence of *Ter1a* or *Ter2b* alone is not restored. These results support our hypothesis and suggest that for all members of the family, the functional protein is an antiparallel dimer (or higher oligomer). Strikingly, this experiment shows that the same function is conserved between distantly related members of the family. This probably reflects the importance of this function in a fundamental cellular process.

In conclusion, our analysis is consistent with an evolutionary path in which a singleton gene encoding a dual-topology protein undergoes gene duplication. The two resulting genes may then evolve in parallel and undergo a drift in the (R+K) bias, leading to a pair of proteins fixed in opposite orientations in the membranes [6]. Alternatively, the two resulting genes can fuse to form a new two-domain protein. Apparently, the selective advantage is in favor of the fusion genes since these forms have spread from prokaryotes to eukaryotes, whereas pairs of duplicated genes are only found amongst cyanobacteria. In both cases, a duplication event provides a fertile ground for evolution. Indeed, the central region, which can exist only in the two-domain proteins, seems to have quickly acquired a proper and supplementary function. The appearance of several acidic residues within the cytosolic region could improve the coordination of divalent cations such as Ca^2+^, providing a better specificity and/or affinity of the protein. This observation tends to strengthen our hypothesis that Ca^2+^ is actually the substrate of the UPF0016 family, or at least some of its members.

### Eukaryotic UPF0016 Members, an Example of Diversification

The two-domain antiparallel eukaryotic members of the family continued to evolve. Even if they share a high degree of conservation, each subfamily delimited on our phylogenetic tree (Fig. 1 & S2) possesses its own features.

Notably, the diversity is the strongest among plant members of the family. Indeed, in each vegetal species, we were able to find between 2 and 5 paralogs, while one single member was present in most other eukaryotic organisms (the splice variants were not taken into account in this study). The abundance of paralogs in plants is a known phenomenon, and the protein diversity in plants is proposed to be generated primarily through gene duplication rather than alternative splicing, as has been proposed for vertebrates [21]–[23]. Directly after their duplication, paralog genes should have the same function. Loss of one of the copies is predicted to be the most favorable outcome. Indeed, only 27% of the genes in *Arabidopsis thaliana* [24] and 16% of the genes in *Saccharomyces cerevisiae* [25] remain duplicated between sister genomic regions originating from duplication events. Fungal members of the subfamily XI are in good agreement with this observation. However, another scenario arises when several copies remain. The selective advantage of such a conservation could be the requirement of several copies for a correct gene dosage or the appearance of divergent functions [23], [26]. In that case, both copies can keep the same function but acquire a complementary pattern of expression/regulation (*subfunctionalization*), or undergo mutations leading to different functions (*neofunctionalization*). The separation of plant and other eukaryotic sequences into subfamilies might be linked to the appearance of new features, putatively leading to new functions. In order to identify these features, several key criteria were analyzed: the length of the N-terminus and the central acidic loop, the conservation of the two canonical motifs, and the predicted topology.

**Figure 5:**
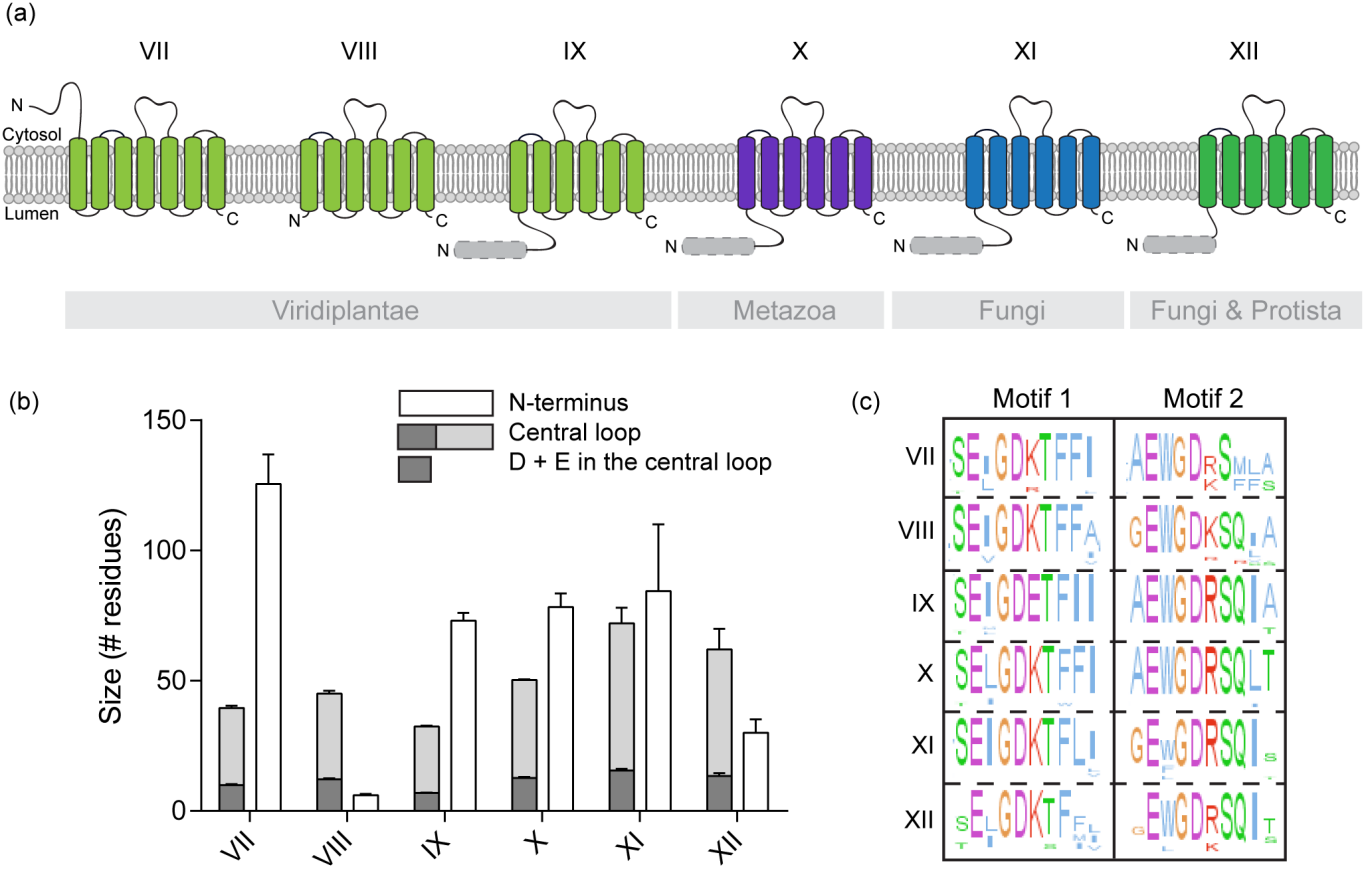
Comparison of the different eukaryotic subfamilies (VII to XII). (**a**) Scheme of the consensus topology for each subfamily, obtained using TMHMM predictions for each sequence. Plant, metazoan, fungal, and protist/fungal subfamilies are colored in light green, purple, blue, and dark green, respectively. When predicted by SignalP 4.0 the signal peptides for most members of the subfamily are depicted as a pale grey dotted box.

Consensus topologies were predicted for each sequence with TMHMM [27] and, when needed, confirmed with Memsat-SMV [28], in order to construct a model corresponding to the topology predicted for most members of each subfamily (Fig. 5a). The arrangement in two clusters of three transmembrane spans surrounding a central cytosolic loop appears to be the general rule for each subfamily, except the plant subfamily VII. In this case, a seventh transmembrane span is predicted just before the previous ones. As a consequence, the N-terminal soluble extension, which usually faces the lumen/outside, is located in the cytosol. According to the hypothesis that the N-terminus is involved in the regulation of the protein activity, this change in the topology could reflect dependence to different regulatory mechanisms. For example, one could imagine that the activation/repression of the protein requires the binding of different regulatory subunits, which, depending on the ortholog, are localized either in the cytosol (subfamily VII) or in the lumen of intracellular compartments (other subfamilies).

The length of the N-terminus is quite variable (Fig. 5a), ranging from no extension to more than 100 residues. The longest N-terminal extension is found in the plant subfamily VII, while the plant subfamily VIII only has a small, if any, extension. This observation suggests that the core and conserved function of the family does not depend on the N-terminal hydrophilic loop. When present, this region could play an accessory and/or regulatory role. These kinds of observations are not unusual. For instance, the plant and animal orthologs of the PMCA (*Plasma Membrane Ca^2+^ ATPases*) family possess a calmodulin-binding autoinhibitory domain which is absent in the yeast protein Pmc1p (calmodulin is a member of the Ca^2+^-binding EF hand superfamily). Similarly, AtCAX1, an *Arabidopsis thaliana* Ca^2+^/H^+^ exchanger member of the cation/Ca^2+^ (CaCA) exchanger superfamily of transporters, possesses a N-terminal autoinhibitory tail which can interact with activator proteins but is absent in its yeast ortholog, Vcx1p [29]. The increase in complexity of these proteins is probably correlated to the increased complexity of higher eukaryotic cells, where the Ca^2+^-signaling pathways have to be regulated with different spatial and temporal properties.

Within the N-terminal region, we identified that cleaved signal peptides were predicted for most members of the subfamilies IX-XII (Table S1), using SignalP 4.1, a bioinformatic tool allowing their prediction with a high degree of confidence [30]. The signal peptides are protein-sorting signals that target proteins for translocation across the endoplasmic reticulum (ER) membrane and regulate membrane insertion efficiencies [31]. Translocation occurs co-translationally through a multiprotein complex called the translocon via a signal recognition particle (SRP)-dependent or a SRP-independent pathway. Recent data suggests that the SRP preferentially binds highly hydrophobic sequences such as transmembrane domains, whereas most signal peptides do not engage SRPs and rather bind to a different set of chaperoning and ER-targeting proteins [32]. Thus, the presence of a signal peptide in some UPF0016 subfamilies might determine their way of insertion in the ER. However, since the presence of a signal peptide does not appear to be the general rule within the UPF0016 proteins, this feature is probably not essential for their insertion in the ER membrane. In the absence of a signal peptide, the first transmembrane domain of the protein is supposed to act as a recognition motif for SRP binding. The different ER-insertion pathways are currently thought to be different means to a similar end, and their respective specificities are not fully understood. These pathways may generate unique microdomains specialized to different protein subpopulations, and confer specific properties (efficiency, suborganellar localization, etc) to the translocation process [32]. The presence or absence of a signal peptide in members of the UPF0016 family could therefore be a supplementary mechanism of regulation ruling the insertion of the different members in the ER membrane.

**Figure 6:**
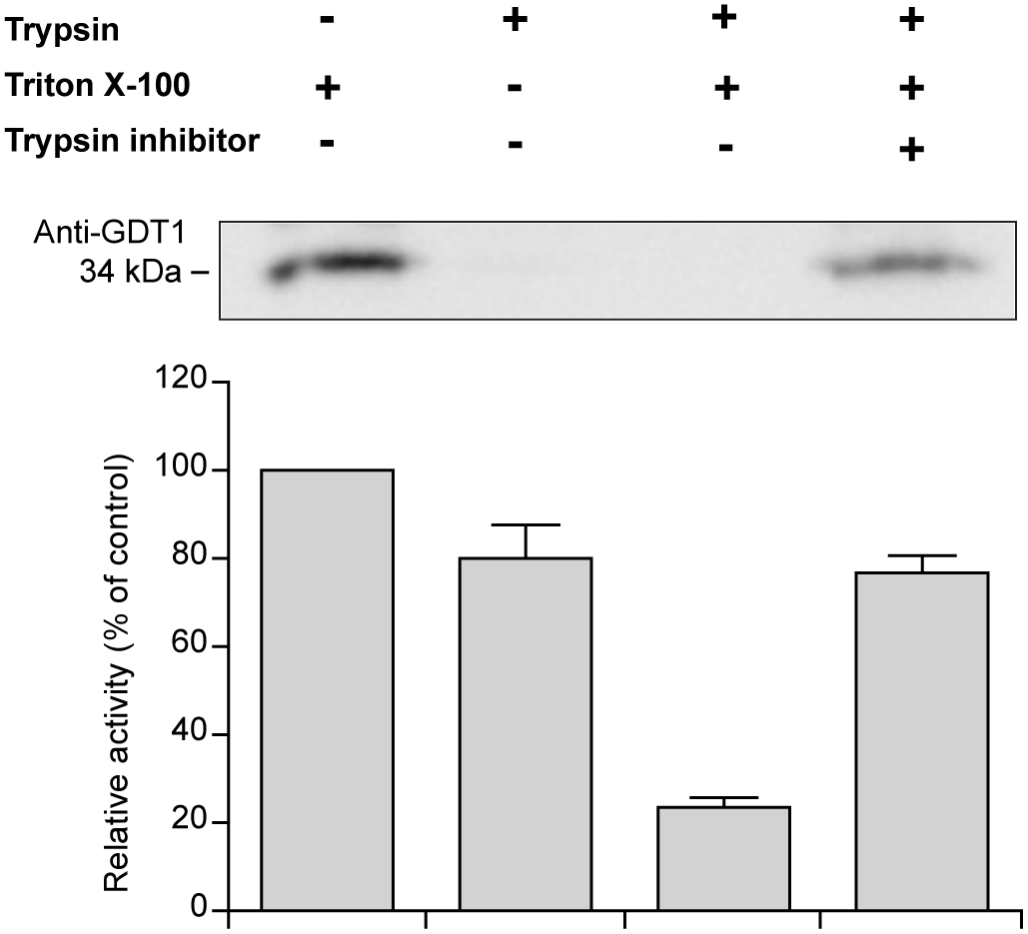
The central loop of Gdt1p is cytosolic. Golgi-enriched fractions obtained by subcellular fractionation on sucrose gradient were submitted to trypsin digestion. In the absence of detergent (Triton X-100), only the cytosolic loops of a membrane protein are accessible to the protease, while the luminal loops are protected against digestion. The integrity of the central loop of Gdt1p after treatment was assessed by immunodetection using a polyclonal antibody raised against this region of the protein (*Top panel*). Measurement of the luminal Golgi-resident GDPase activity under the same conditions was used as a control to confirm the integrity of our vesicles (*Bottom chart*). Results are mean ± SEM from 4 independent experiments.

The length of the central loops is less variable and, although they can differ in their sequences, they all possess roughly the same ratio of negatively charged acidic residues (Fig. 5b). As a first step to confirm the predicted topology of the members of the family, we demonstrated experimentally that the central loop of Gdt1p is cytosolic (Fig. 6). Golgi vesicles purified by sucrose gradient were treated with trypsin in the presence or absence of detergent (Triton X-100) and analyzed by immunodetection using a polyclonal antibody raised against the central loop of Gdt1p. Unlike the luminal Golgi-resident GDPase which was protected from digestion in the absence of the detergent, the central loop of Gdt1p was efficiently digested by the protease, confirming its cytosolic localization.

As previously stated, the presence of such an acidic motif is typical of the Ca^2+^-binding proteins [33] or Ca^2+^ transporters. We previously discussed the striking similarities which exist between UPF0016 proteins and members of the CaCA superfamily, although they do not share any notable sequence homology [3]. The first similarity concerns their topology: they are all made of two homologous hydrophobic domains separated by a central loop rich in acidic residues [8]. Interestingly, the crystal structure of a member of this superfamily, Vcx1p, the yeast vacuolar Ca^2+^/H^+^ exchanger, has been determined recently and shed light on the mechanistic relevance of some motifs and domains [34]. According to the authors, the acidic motif localized in the central loop of Vcx1p lies across its cytosolic entrance and coordinates two Ca^2+^ ions. They suggest that this acidic loop maintains an α-helical conformation in the presence of the two coordinated Ca^2+^ ions, and becomes more flexible in their absence. This conformational change indicates a possible Ca^2+^-dependent regulatory function for this region. The resemblance with the UPF0016 family members is striking and tends to prove that their central acidic loop might also exert a regulatory function through the coordination of Ca^2+^.

Furthermore, the two homologous signature sequences of Vcx1p (G-N-x-x-E) might also be compared to the E-Φ-G-D-(KR)-(TS) motifs of the UPF0016 family. These sequences form kinks in two transmembrane helices and meet in the mid-membrane plane, forming the Ca^2+^-binding active site of Vcx1p [34]. In that site, the cation is coordinated by a glutamate and a neighboring serine, together with three ordered water molecules, themselves coordinated by a glutamate, an asparagine, and the backbone carbonyl of a glycine. Those key residues are strikingly similar to those found in the UPF0016 family consensus motifs.

An exception exists among the eukaryotic members of the family. The first motif of the plant subfamily IX shows an E-Φ-G-D-**E**-T sequence instead of the consensus sequence (Fig. 5c). This difference leads to the substitution of a positively charged lysine with a negatively charged glutamate, and must therefore affect the activity of the proteins. The formation of an even more negative microenvironment could shift the affinity of the protein towards other substrates, putatively ions with a greater valency (*e.g.* Fe^3+^).

Obviously, we think that these similarities are not insignificant, and could be an example of convergent evolution. Together with our previous experimental results [2], [3], this analysis lead us to the hypothesis that the UPF0016 family is a new group of cation/Ca^2+^ exchangers whose function is highly conserved through evolution. This family and the CaCA superfamily are not to be confused or merged because, despite functional and structural similarities, they do not share any notable primary sequence homology.

## Materials and Methods

### Bioinformatic Analysis

The phylogenetic tree was built with the MEGA5 [35] software. Briefly, the sequences were acquired using a psiBLAST algorithm against the GDT1 (YBR187w) sequence and by selecting as much organism variety as possible among the fully sequenced genomes (NCBI genome database: http://www.ncbi.nlm.nih.gov/genome/browse/, consulted in December 2013). The sequences were then aligned with MUSCLE [36] using default parameters and the neighbor-joining (NJ) method was used to determine the phylogenetic relationship existing between the sequences. The reliability of each internal branch in the resulting trees was supported with 1000 bootstrap resampling (only values higher than 50 are indicated on the tree).

### Yeast Strains, Plasmids, and Culture Media

The yeast strains used are BY4741 (from Euroscarf) and BY4741 *pmr1Δ/gdt1Δ* (*Mata his3Δ1 leu2Δ0 ura3Δ0 lys2Δ0 met15Δ0 gdt1::KanMX4 pmr1::KanMX4*) [3]. The plasmids are derived either from the pRS315 or pRS316 vectors. The TPI (triose phosphate isomerase) promoter is inserted between SacI and NotI, the gene of interest between NotI and SpeI, and the CYC1 (cytochrome C) terminator between SpeI and HindIII restriction sites in the multi-cloning site of the vectors. The bacterial genes (Ter1a, Ter2b, and Dma) were synthesized by GenScript, after application of their codon optimization tool for yeast and bacteria. The inserts were then amplified by PCR with the FastStart High Fidelity PCR system (Roche). All of the restriction enzymes were from New England Biolabs and the T4 DNA ligase from Promega. Non transformed yeast cells were routinely cultured at 28°C in YD medium (2% yeast extract KAT, 2% glucose). Cells transformed with plasmids were grown in SD minimal medium (0.7% yeast nitrogen bases without amino acids (Difco), 2% glucose, supplemented with all amino acids except those used as selection markers for plasmid maintenance). When indicated Ca^2+^ chloride salt (500 mM) was added after sterilization. In that case, the SD composition changed slightly (0.2% yeast nitrogen base without amino acids and ammonium source (Difco), 0.4% NH_4_Cl, 2% glucose).

### Yeast Growth Assays

For the drop test, cells were precultured overnight in 5 ml of SD without Ura and Leu. The cultures were then diluted to an OD_600_ of 0.3 and three tenfold dilutions were spotted onto the solid medium (addition of 2% agar) supplemented with CaCl_2_. The plates were incubated at 28°C for 4–10 days and monitored daily.

### Trypsin Digestion Assay

Golgi vesicles were purified by subcellular fractionation on sucrose gradient as described previously [3]. Fractions enriched in Gdt1p were submitted to trypsin digestion. For this purpose, 35 µl of Golgi-enriched fractions were incubated for 1 h at 25°C in a final volume of 50 µl containing 0.1 mg/ml of trypsin. When needed, Triton X-100 was added to a 1% (v/v) final concentration. For the negative control, the Golgi-enriched fractions were pre-incubated for 10 min at room temperature with a trypsin inhibitor mixture (10 mM PMSF, 0.05 mg/ml trypsin inhibitor (Type I-S; Sigma) and a protease inhibitor cocktail (PIC; Sigma) containing a final concentration of 8 µg/ml leupeptin, aprotinin, antipain, pepstatin, and chymostatin). At the end of the all experiments, trypsin digestion was stopped by addition of the trypsin inhibitor mixture for 10 min, followed by addition of Triton X-100 to a 1% (v/v) final concentration. The resulting samples were analyzed by SDS/PAGE and Western Blotting using a rabbit polyclonal antibody raised against the central loop of Gdt1p (residues 119–185) [3].

GDPase activity was tested as described in [37]. Briefly, the GDPase reaction was initiated by the addition of 10 µl of the Golgi-enriched fraction to 90 µl of reaction mixture (20 mM imidazole, 2 mM CaCl_2_, 2 mM GDP, pH 7.4), and performed at 30°C for 20 min. The reaction was stopped by the addition of 300 µl of SDS 1%. The inorganic phosphate produced by the reaction was then dosed by a colorimetric reaction: 400 µl of a molybdate solution (50 g/L (NH_4_)_6_Mo_7_O_24_.4H_2_O, 4N H_2_SO_4_) and 400 µl of an Elon solution (10 g/L Elon (Kodak), 30 g/L Na_2_S_2_O_3_) were added, and the absorbance was measured at 700 nm after 15 min of incubation at room temperature.

## Supporting Information

Figure S1 Phylogenetic tree of the prokaryotic members of the UPF0016 family. The tree was constructed using the neighbor-joining method. It is drawn to scale with branch lengths measured as number of substitutions per site. Different taxonomic groups are represented by different colors, while different UPF0016 subfamilies are delimited by grey areas and numbered from I to VI. Bootstrap values (after 1000 iterations) higher than 50 are indicated.
(TIF)

Figure S2 Phylogenetic tree of the eukaryotic members of the UPF0016 family. The tree was constructed using the neighbor-joining method. It is drawn to scale with branch lengths measured as number of substitutions per site. Different taxonomic groups are represented by different colors, while different UPF0016 subfamilies are delimited by grey areas and numbered from VII to XII. Bootstrap values (after 1000 iterations) higher than 50 are indicated.
(TIF)

Figure S3 Multiple alignment of the members of the UPF0016 family. One member of each subfamily was randomly selected and aligned with its orthologs using the Muscle algorithm. The resulting alignment was visualized in Jalview: residues are shaded in blue depending on their conservation. The quality score is inversely proportional to the average cost of all pairs of mutations observed in a particular column. The consensus line represents the HMM logo view of the alignment. Putative transmembrane spans were predicted directly from the alignment file using TMAP and are depicted above the sequence (TM1 to TM6).
(TIF)

Table S1 List of the 149 protein sequences used for this work. Abbreviation, NCBI accession number, scientific name, taxonomic groups and subgroups of the key organisms as classified in the NCBI genome database. For prokaryotic members of the family, the repartition into singleton, paired, or fusion gene is specified. For eukaryotic members of the family, the presence (Yes) or the absence (No) of a signal peptide was predicted using SignalP 4.1.
(XLSX)
