# Supplementary figures and images for: Molecular Evolution of a Novel Family of Putative Calcium Transporters
Source: PLoS One. 2014 Jun 23;9(6):e100851. doi: 10.1371/journal.pone.0100851 (PMC4067407; doi:10.1371/journal.pone.0100851)

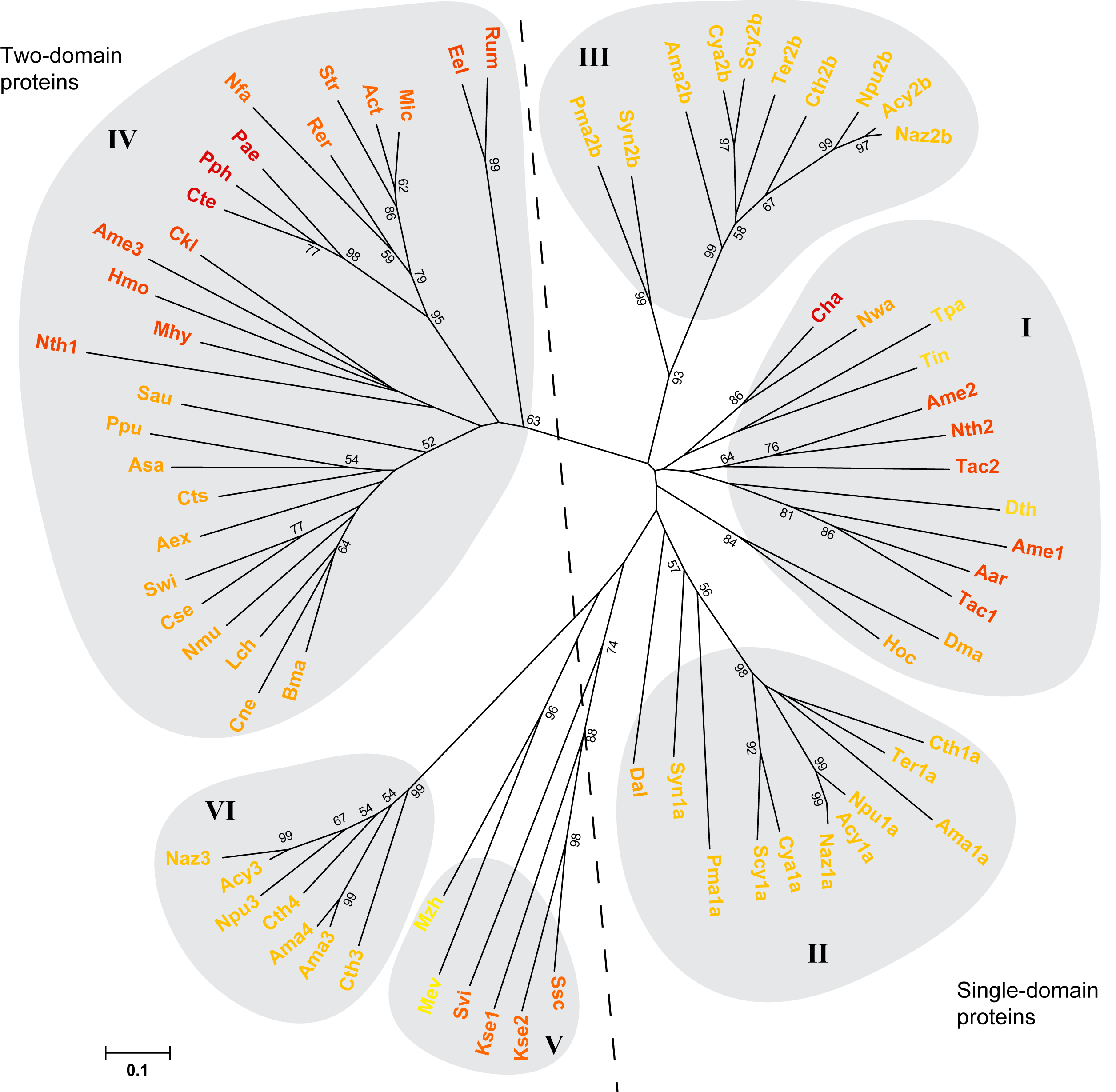

Supplement: Figure S1 — Phylogenetic tree of the prokaryotic members of the UPF0016 family. The tree was constructed using the neighbor-joining method. It is drawn to scale with branch lengths measured as number of substitutions per site. Different taxonomic groups are represented by different colors, while different UPF0016 subfamilies are delimited by grey areas and numbered from I to VI. Bootstrap values (after 1000 iterations) higher than 50 are indicated. (TIF) [file pone.0100851.s001.tif]

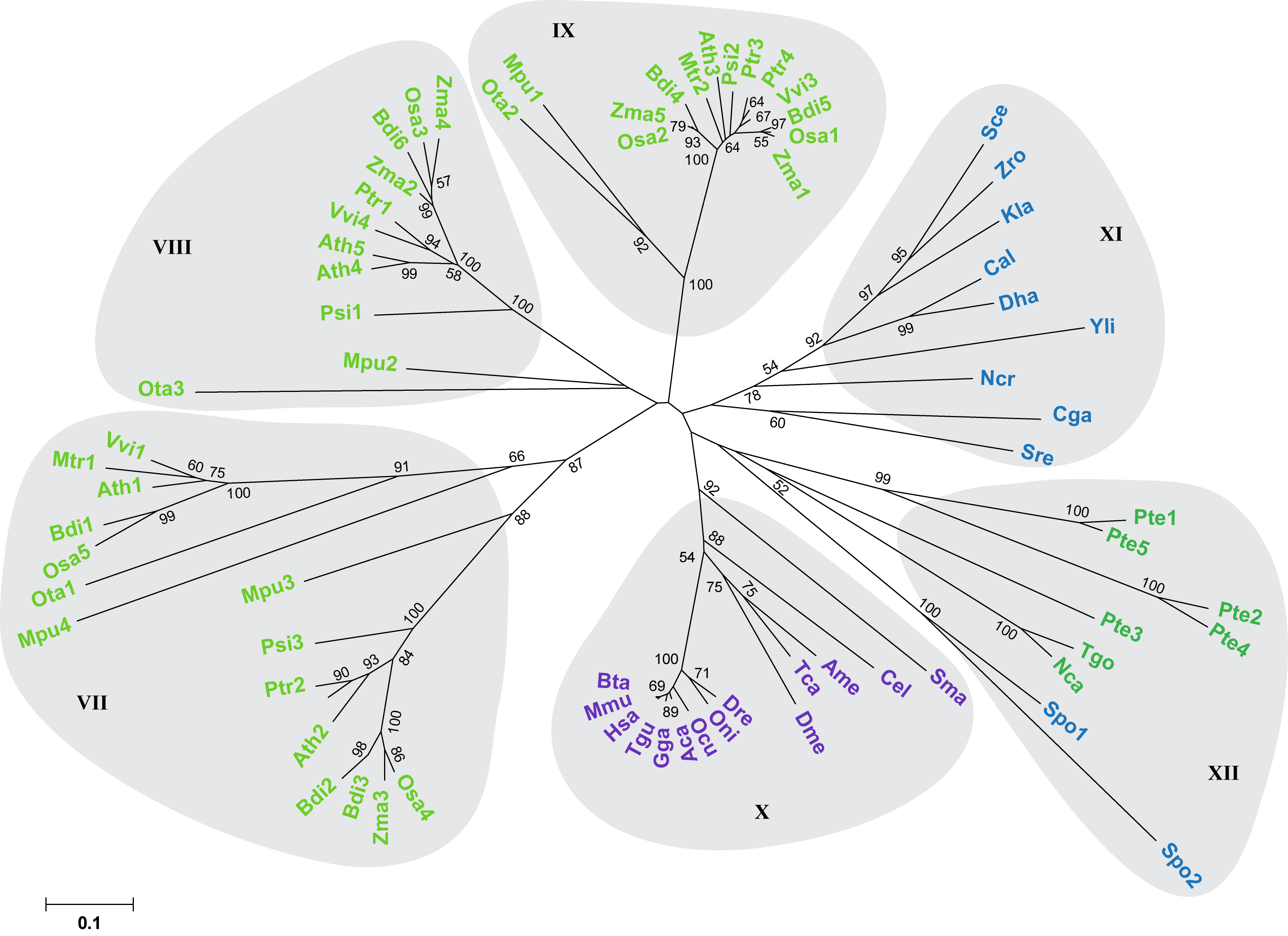

Supplement: Figure S2 — Phylogenetic tree of the eukaryotic members of the UPF0016 family. The tree was constructed using the neighbor-joining method. It is drawn to scale with branch lengths measured as number of substitutions per site. Different taxonomic groups are represented by different colors, while different UPF0016 subfamilies are delimited by grey areas and numbered from VII to XII. Bootstrap values (after 1000 iterations) higher than 50 are indicated. (TIF) [file pone.0100851.s002.tif]

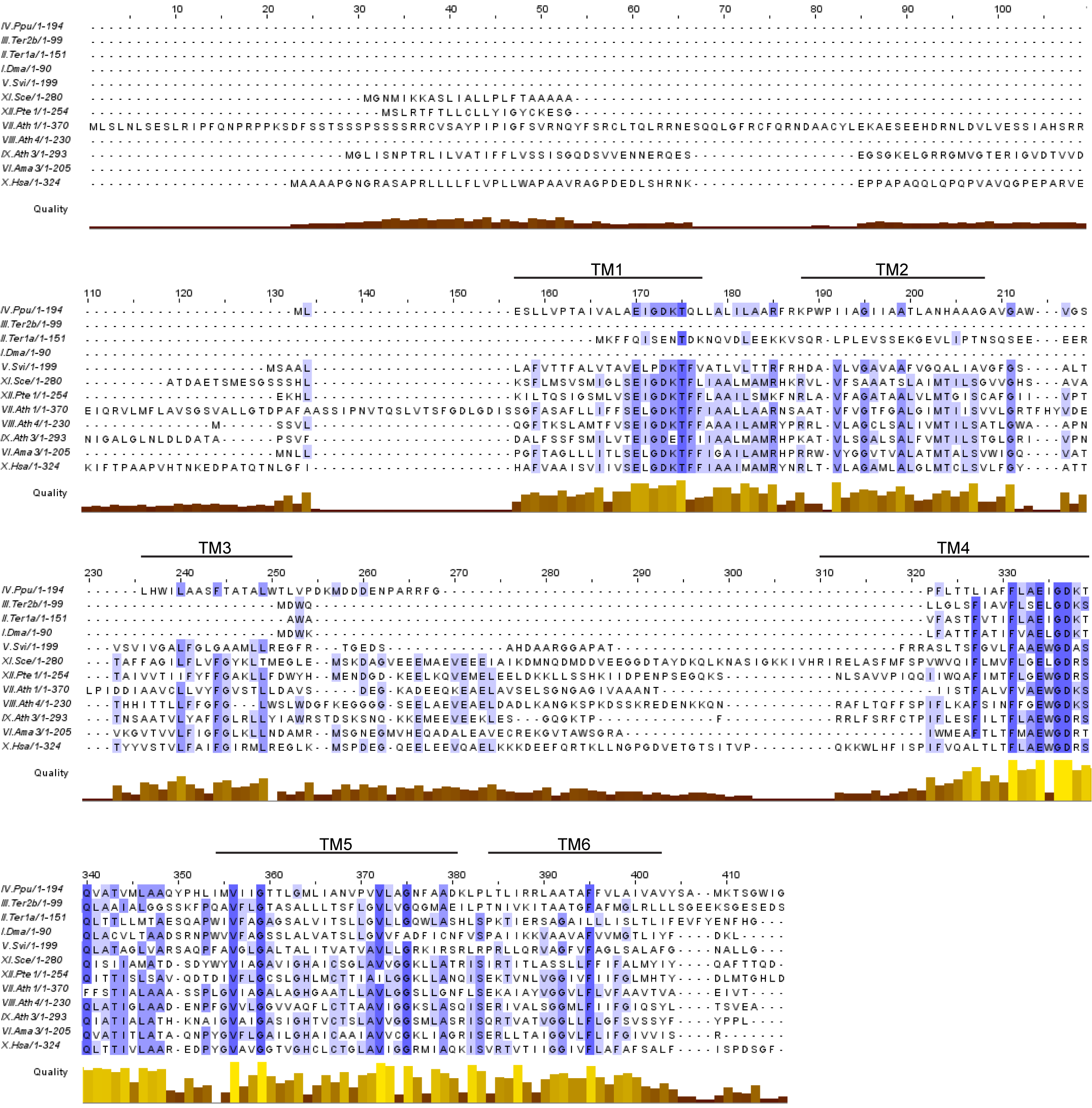

Supplement: Figure S3 — Multiple alignment of the members of the UPF0016 family. One member of each subfamily was randomly selected and aligned with its orthologs using the Muscle algorithm. The resulting alignment was visualized in Jalview: residues are shaded in blue depending on their conservation. The quality score is inversely proportional to the average cost of all pairs of mutations observed in a particular column. The consensus line represents the HMM logo view of the alignment. Putative transmembrane spans were predicted directly from the alignment file using TMAP and are depicted above the sequence (TM1 to TM6). (TIF) [file pone.0100851.s003.tif]
